# Sensitivity to Sunk Costs Depends on Attention to the Delay

**DOI:** 10.3389/fpsyg.2021.604843

**Published:** 2021-02-22

**Authors:** Rebecca Kazinka, Angus W. MacDonald, A. David Redish

**Affiliations:** ^1^Graduate Program in Clinical Science and Psychopathology Research, University of Minnesota, Minneapolis, MN, United States; ^2^Psychology Department, University of Minnesota, Minneapolis, MN, United States; ^3^Neuroscience Department, University of Minnesota, Minneapolis, MN, United States

**Keywords:** mTurk, sunk costs, attention, foraging, multiple decision systems, cognition, effort

## Abstract

In the WebSurf task, humans forage for videos paying costs in terms of wait times on a time-limited task. A variant of the task in which demands during the wait time were manipulated revealed the role of attention in susceptibility to sunk costs. Consistent with parallel tasks in rodents, previous studies have found that humans (undergraduates measured in lab) preferred shorter delays, but waited longer for more preferred videos, suggesting that they were treating the delays economically. In an Amazon Mechanical Turk (mTurk) sample, we replicated these predicted economic behaviors for a majority of participants. In the lab, participants showed susceptibility to sunk costs in this task, basing their decisions in part on time they have already waited, which we also observed in the subset of the mTurk sample that behaved economically. In another version of the task, we added an attention check to the wait phase of the delay. While that attention check further increased the proportion of subjects with predicted economic behaviors, it also removed the susceptibility to sunk costs. These findings have important implications for understanding how cognitive processes, such as the deployment of attention, are key to driving re-evaluation and susceptibility to sunk costs.

## Introduction

Sunk costs are costs that have been already been spent and cannot be recovered. The sunk cost fallacy is a decision bias in which individuals continue to invest money, time, or energy into a bad deal because of the effort that they have already put into it. Including sunk costs in one’s decision is considered irrational, as all future outcomes include the same sunk costs, and thus decisions should be made based on future expectations. However, there are many cases where humans include sunk costs in their decisions. For example, an individual may be more unlikely to quit after an investment decision even when negative outcomes appear, leading to inappropriate escalation (Staw, 1976; Staw and Fox, 1977; Staw and Ross, 1989; Mcafee et al., 2010). Recent studies have found that with naturalistic tasks the sunk cost fallacy appears in mice and rats as well. These behaviors can even be compared directly to humans in a translational pair of tasks, Restaurant Row (rodent) and the WebSurf Task (human; Sweis et al., 2018a).

**Figure 1:**
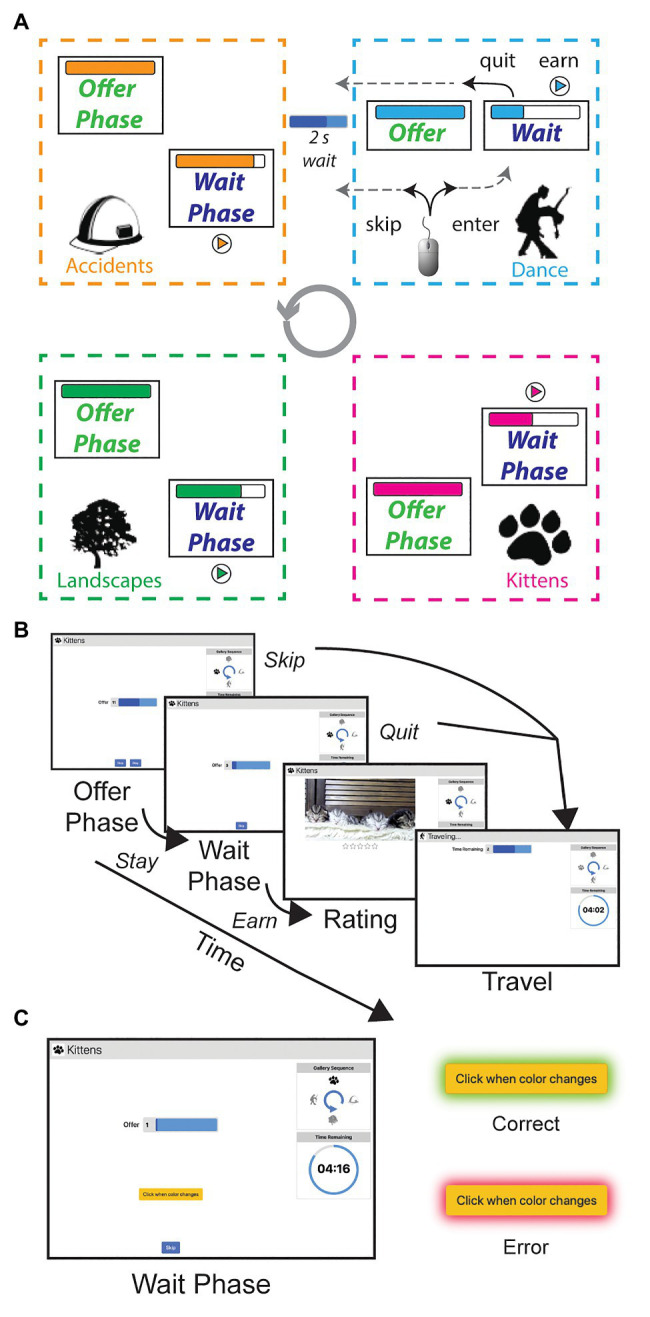
Diagram of the task. **(A,B)** Participants foraged for different categories of video clips. Categories (Landscape, Dance, Bike Accidents, Kittens) were in a set order randomized across participants. Each trial began with an offer phase, where they were shown a delay and could decide to skip or stay. If they stayed, they entered the wait phase and the delay timer began counting down. At this time they could choose to quit the trial. If they earned the trial, they watched a 4s clip related to the category, and then rated it on a five-star scale. If a participant chose to skip, quit, or complete the trial, they all end with a travel time of 2s before starting a new trial with a new delay and category. **(C)** The attention check version was the same as the original version, except for one change in the Wait Phase. A yellow button appeared, which changed to a darker yellow every 7–10s. Participants had to click the button within 2s of the color change to show they were paying attention. If they correctly responded, the button border turned green; if they missed the button or pressed it outside of the color change, it would turn red.

In such naturalistic tasks with rodents and humans, sensitivity to sunk costs can be measured as the increasing willingness to accept an offer as a function of the effort already spent. In the WebSurf Task, individuals had 30min to forage for video clips from four different categories, which were presented in a fixed order. For each trial, participants were shown a “download bar” – an offered delay that they had to wait through in order to watch a 4s video clip from a known category. Each trial consisted of two phases: an offer phase and a wait phase. On entering the offer phase, they were informed of the delay that would be required before the video would be shown. Importantly, the delay did not count down (the download bar remained “full”) while the subject was in the offer phase. The subject could choose to reject the offer (skip), proceeding on to the next category with a new delay, or accept the offer (enter), proceeding into a waiting phase during which the download bar began shrinking as the delay counted down. There was no time limit for the subject during the offer phase. While waiting in the waiting phase, they could still quit and move on to a new trial without receiving the reward. If they waited through the countdown until the download bar emptied completely, the 4s video would play after which they rated the video (1–5 stars). Then, they would move on to a new trial with a different category (Figure 1A). This task, along with its similar rodent translation (in which rodents forage for flavored food pellets), provides an opportunity to measure the influence of sunk costs on decisions by measuring the likelihood of quitting from the wait phase (or skipping from the offer phase) as a function of the time spent in each phase. Previous studies have found that subjects (mice, rats, undergraduates) show an influence of sunk costs in making quit decisions in the wait phase, but not in making skip decisions in the offer phase (Sweis et al., 2018a).

There are a number of theories that can produce this relationship between effort spent and willingness to accept an offer, including that the effort spent decreases energy stores, which increases the value of the reward (Pompilio et al., 2006; Aw et al., 2011), that proximity to the goal increases the value non-linearly (Singer and Zentall, 2011), or that internal algorithm can more easily assess prior effort and reward than predicting future outcomes, and therefore animals may plan based on prior knowledge rather than updating for the present (Wikenheiser et al., 2013). However, these theories all suggest that sunk costs should be a continuous function of the effort spent, and should occur even when an individual has not committed to a choice. These theories would imply sunk time into a decision should influence choices in both the offer and wait phases, inconsistent with the observations of Sweis et al. (2018a).

In Sweis et al. (2018a), the sensitivity to sunk costs in mice, rats, and humans depended on the context of the situation. This sensitivity did not appear when subjects were making decisions in a separated “offer phase”; sunk costs only began to accrue after subjects entered the “wait phase” and began waiting for the actual offer. The distinction between deliberating the offer and deciding to opt out of an already-initiated task may indicate crossing a “mental Rubicon” – switching from a deliberative state towards one more focused on carrying out the task (called “implemental” by Gollwitzer et al., 1990). Named after Caesar’s decision to cross the Rubicon River into Rome which violated a norm that could not be undone (Suetonius, 121/1957), this literature suggests that decision points and commitment affect a person’s ability to assess new information (Heckhausen and Gollwitzer, 1987). Importantly, while deliberating, an individual is contemplating the pros and cons of an option, and is more receptive to all kinds of information; in the implemental state they become goal-oriented and become less attentive to new information (see Gollwitzer, 2012; Achtziger and Gollwitzer, 2018 for overview). Recent studies in this area of research have found that individuals who are manipulated into a deliberative mindset were slower in their decision making in a gamble (Ludwig et al., 2020), while those in an implemental mindset were better suited to learning during a reinforcement learning task (Li et al., 2019). No prior research in mindset theory has specifically tested sunk cost sensitivity, but it is possible that these states may also inform decisions to quit after re-evaluating less valuable options.

The hypothesis that the decreased likelihood of quitting from the wait phase as a function of time spent arises from a sensitivity to sunk costs implies a requirement that the subject be cognizant of the delays. We, therefore, set out to test the necessity of cognitive processes for attending to the decision in the sunk cost fallacy by recruiting participants on Amazon Mechanical Turk (mTurk) to perform the WebSurf task with two different variants. The original version of the task matched the one described above (allowing a replication of a population beyond undergraduates and online rather than in-person). The second version included an “attention check” to ensure that subjects remained attentive to the task during the delay (Figure 1A). Although we originally intended for the “attention check” to ensure that subjects did not divert themselves during the experiment, we found that it also distracted them during the delay and changed behavior. As such, for clarity, we will refer to this second version of this task as the “distractor version” in the rest of the manuscript. In the distractor version of this task, we included a simple task during the wait phase, in which participants needed to click a button when it changed to a new luminance every 7–10s. This addition allowed us to test if the participant was engaged with the task. The naturalistic component of this task allows for measurement of sunk cost under the manipulation of the environment of the task. We found that this simple addition impacted task engagement and performance, and that it eliminated the susceptibility to sunk costs.

## Materials and Methods

### Participants

One thousand two hundred thirty individuals were recruited through Amazon mTurk. Of those, 1,114 completed a questionnaire providing demographic information [48.6% male, 50.8% female, 0.6% nonbinary; mean age = 37.4 (11.5) years old; 74.9% White, 10.1% Black/African American, 7.3% mixed/other, 6.4% Asian, 0.8% Alaskan/American Indian, <1% Pacific Islander, 0.5% refused; 85.1% non-Hispanic or Latinx, 12.8% Hispanic or Latinx, 2.1% refused]. They completed one of two different versions of the task – either the original version or the distractor version.

Because there is a known problem with computer-controlled (non-human) bots completing Amazon mTurk Human Intelligence Tasks (HITs), we used several exclusion criteria to screen participants, described in procedures. The exclusions we used for bots were planned before we started collecting experimental data. Subjects who were identified as likely bots were excluded before they were able to complete the WebSurf task. Additionally, once started, participants had to get to the end of the task in 30min and provide rankings for each category. Finally, if they had fewer than 40 trials, we did not include their responses, as we could not adequately estimate their thresholds (see analyses for threshold explanation). Six hundred fifty-one agents started the original version. After applying the exclusion criteria and removing agents unlikely to be actual humans and agents that did not finish the task, we were able to use data from 259 participants (47% male, 53% female, <1% nonbinary; mean age = 36.6, SD = 11.6; 73.4% White, 9.6% Black/African American, 10.0% mixed/other, 7.0% Asian, 0% Alaskan/American Indian, 0% Pacific Islander, 0% refused; 90.0% non-Hispanic or Latinx, 8.9% Hispanic or Latinx, 1.1% refused). Five hundred seventy-nine individuals started the distractor version, and, after applying our exclusion criteria, we were able to use data from 280 participants (50% male, 49% female, 1% nonbinary, mean age = 38.1, SD = 11.3; 76.8% White, 12.1% Black/African American, 5.0% Asian, 4.3% mixed/other, 1.1% Alaskan/American Indian, 0% Pacific Islander, 0.7% refused; 91.4% non-Hispanic or Latinx, 7.2% Hispanic or Latinx, 1.4% refused). Our rate of data that are likely bots is consistent with previous work (Chmielewski and Kucker, 2020). See Supplemental Table S1 for a breakdown of demographics. All participants consented to participate, and all methods were approved by the University of Minnesota Institutional Review Board.

### Task

The WebSurf task (Abram et al., 2016) was originally based on the Restaurant Row task, which is a foraging task designed for rodents (Steiner and Redish, 2014; Sweis et al., 2018d). In the original version of the task, individuals were told they could spend 30min to “surf the web” for 4-s videos in four categories: Bike Accidents, Landscapes, Dance, and Kittens. We refer to the four different video sites as “galleries.” Each trial consisted of an offer phase, a potential waiting phase, and a potential earning phase. Each trial began with entry into the offer phase, wherein a subject was told the category and an amount of time they would have to wait before they could watch the video clip. Offers ranged between 2 and 30s and were randomized without replacement until all values for each category had been offered, in which they began a new sequence of randomization without replacement. In the offer phase, subjects were given the choice to enter and wait out the time delay, or skip and continue to the next category with a new time delay. Importantly, the download bar did not move (diminish, count down) during the offer phase. There was no time limit in the offer phase. If they decided to enter the wait phase, the delay began counting down. Through the countdown, subjects were given the option to quit if they no longer wished to wait for the video. If they completed the wait through the countdown, the video played and subjects were asked to rate it on a five-star scale. Skipping, quitting, or completing the trial led to a 2-s delay to travel to the next trial (see Figure 1). Trials continued in this manner until the 30min had completed. Participants saw the time left in the 30min prominently on the screen. At the end of the 30min, they were asked to rank the four categories. Categories appeared in a fixed order for each individual that was randomized between individuals.

The distractor version added a yellow button during the wait phase. This button changed from lighter to darker yellow every 7–10s. After changing, participants had 2s to respond. The button border turned green if they responded in time, and turned red if they missed the button or pressed it anytime outside the response window. The button color reset to yellow after feedback was provided. There were no explicit consequences for failing to engage with the wait phase task; however, workers were encouraged to do a good job.

### Procedure

Individuals were recruited through Amazon mTurk. Subjects were first informed of the risks and benefits of participation per the University of Minnesota Institutional Review Board and consented to participate. Participants were required to have completed at least 50 successful HITs, have an approval rate of 95%, and be located in the United States to be able to begin the HIT. The session began with a reCAPTCHA v2 (Google, 2014), which is a system designed to detect whether or not the actor is a human or not (von Ahn et al., 2003); if subjects failed the reCAPTCHA, they were not allowed to continue and were not paid. They were offered $1.00 for completion of a set of questionnaires hosted through Qualtrics (Qualtrics, 2005), including the Infrequency Scale (Chapman and Chapman, 1983), which contains questions about highly improbable situations. We used this scale as an additional screen for bots; if respondents endorsed more than two of these improbable situations, they were not allowed to continue with the task, but were paid the $1.00. After ~20min of questionnaires, participants completed a short colorblind vision test, using the Ishihara test. We did not use this for any additional screening. They then read through a short set of instructions for the task and watched one version of each video category before beginning the task. The task ran for 30min, without interruption. Several measures were put in place to ensure engagement. All countdown measures paused if the window was switched to a new tab. Furthermore, inactivity for more than 5min led to discontinuation of the task. Individuals who completed all parts of the study were paid $5.00 for their time. Total time to complete was ~45–65min, including time for the questionnaire, Ishihara test, and instructions for the task. Participants who partially completed the task were paid for their time based on a prorated hourly rate of $5/h.

### Analyses

Analyses were conducted using MATLAB 2018b on MacOS (MathWorks; MATLAB, 2018). Data were sorted into trials, beginning at the offer phase of each gallery. Each trial recorded whether or not participants chose to skip or stay, and if they stayed, quit, or earn. Trials, where they did not enter the wait phase, were not included in the calculation of the percentage of quits. Percentages of stay and quit choices were calculated for each individual.

According to Marginal Value Theorem, in making an exploration/exploitation decision, there is a rate of reward average that exists broadly in the world that individuals are trying to estimate for themselves (Charnov, 1976). Individuals make decisions to stay or leave a patch in comparison to the estimated world rate of reward, i.e., staying if the current patch is greater, and leaving when the current patch becomes worse. Therefore, the relative difference is an important metric when deciding whether to stay or leave. When applied to the WebSurf task, we can hypothesize that participants assume that the videos have an average value (averaged over all videos) and a distribution of known costs (uniform distribution of 2–30s), allowing for a stable expected rate of reward. We can assume that decisions within the offer phase are based on a comparison of the costs of the video and the expected reward of the value of the videos within that gallery against the expected costs (2–30s) and value of the next video. Standard decision theory predicts that this should produce a threshold decision – for delays below the threshold, the subject should stay, while for delays above the threshold, the subject should skip. Of course, once the subject has made a choice, we would not expect them to re-evaluate that decision; however, we find that these theories do not entirely explain behavior.

We estimated the threshold offer amount where individuals changed behavior from mostly enter to mostly skip. We expected individuals to skip longer offers and stay for shorter ones. Thresholds were calculated using a Heaviside function to fit the best value that identified the fewest number of error trials using a maximum likelihood. Error trials are identified as being choices that did not match the expected behavior for the threshold, i.e., skipping a trial below the threshold (one that should be valuable) or staying for a trial above the threshold (one that should not be valuable). We additionally ran this analysis from lowest to highest and highest to lowest offers to identify “inverted” subjects, in which individuals tended to stay for longer offers and skipped shorter ones (contrary to expectations), see Supplemental Figure S1. We ran this analysis both for the offer phase (described above), and the wait phase, in which trials which the subject quit from were compared to trials in which the subject waited out the full delay to “earn” the video. To test if thresholds reflected other subjective preferences such as average ratings and final rankings of the galleries, we correlated these three categories for each individual. For some individuals, we were unable to calculate all of the correlations, and therefore they were removed from a one-sample t-test to compare the sample to zero; degrees of freedom for each of these analyses varied slightly.

Analyses between the two conditions, the original and distractor versions, were conducted using two-tailed *t*-tests or ANCOVAs, as appropriate. Sunk cost was calculated by binning every choice by the amount of time spent and time remaining in the trial across all participants based on trials where they entered the wait phase. For each bin, we calculated the percentage of those trials which were ultimately earned out of all trials in that bin. A two-way ANCOVA (time spent and time remaining in individual trials as continuous variables) calculated the significance of the relationship between time spent and time remaining in the given trial and their effects on the probability of earning the video. To this end, we conducted our sunk cost analyses such that *N* was based on the number of bins included. Note, for this purpose the primary variable of interest is whether the attention check is included, and no further individual difference variables are examined in this analysis. We used the MATLAB function anovan to calculate the ANCOVA analyses which allowed *time spent* and *time remaining* to be treated as continuous variables.

We also individually compared each non-zero Time Spent bin against the 0-s Time Spent to measure the impact of time spent. However, the number of data points available for each bin varied due to offer length; for example, there were far more trials in the 0-s time remaining bin (all trials) than those with 20-s spent, as fewer trial offers were over 20s long (only trials with 21–30s offers could be used to determine the effect of waiting 20s on decisions). To correct for this issue, we compared each bin to the same trials when 0s were spent. This required adjustment in the number of trials included in the 0s comparison depending on the amount of Time Spent that was being compared, i.e., truncating the 0s Time Spent group to the max Time Remaining available for the corresponding bin. This method allowed for equal comparison to baseline (0s) for each bin. See Sweis et al. (2018a) for more detail. A two-way ANCOVA (Time Spent and Time Remaining) was then again used to compare the significance between the observed values and the adjusted control. Bonferroni correction was applied when testing the significance. To calculate the slope of the relationship between time spent and time remaining at the selected integers for time spent, a linear regression was applied.

## Results

**Figure 2:**
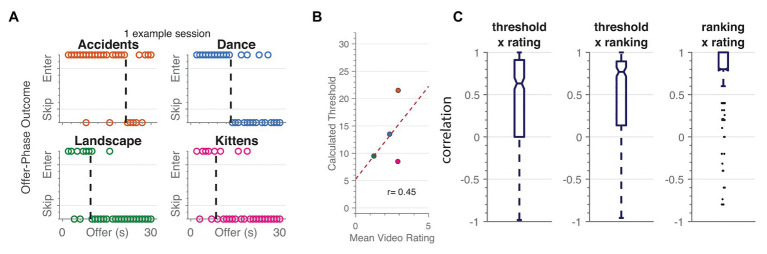
Replication of basic behavioral patterns in WebSurf among the mTurk population. **(A,B,C)** We found a similar pattern of results to previous versions of the task, in which thresholds reveal preferences for different video galleries **(A)**. These thresholds were correlated with average ratings of the videos at an individual level **(B)**, and the overall group showed positive relationships between thresholds, ratings, and rankings **(C)**. * <0.05, ** <0.01, *** <0.001.

Data collected from Amazon mTurk users on the original version of WebSurf replicated findings of data collected in the lab (Abram et al., 2016, 2019a; Sweis et al., 2018a), as well as to rodents on Restaurant Row (Steiner and Redish, 2014; Sweis et al., 2018a). The majority of individuals stayed for shorter delays and skipped longer delays (93.9% across both samples). Thresholds fit by a Heaviside function for each category were correlated with average ratings and final rankings, thus replicating findings that greater thresholds represent revealed preferences for each category [one-sample *t*-test. Threshold × rating: mean *r* = 0.42, 95% CI (0.37 0.47), *t*(471) = 15.5, *p* < 0.001, *d* = 0.71; threshold × ranking: mean *r* = 0.46, 95% CI (0.41 0.51), *t*(490) = 17.8, *p* < 0.001, *d* = 0.79; ranking × rating: mean *r* = 0.79, 95% CI (0.76 0.82), *t*(485) = 57.3, *p* < 0.001, *d* = 2.63; Figures 2A,B,C]. Overall, average ratings for each category had a mean of 2.7 [95% CI (2.6 2.8)].

However, a small proportion of individuals (6.1%; 33 participants) made “inverted” choices, i.e., they stayed for the longer delays and skipped the shorter delays. Thresholds of inverted-choice individuals were negatively correlated with average rankings for each category and marginally negatively correlated with ratings, which suggests that the choices still revealed preferences, but in an inverted manner (Supplemental Figure S1). Rankings and average ratings were still highly correlated [one-sample *t*-test. Threshold × rating: mean *r* = −0.19, 95% CI (−0.40 0.009), *t*(32) = 1.95, *p* = 0.060, *d* = 0.33; threshold × ranking: mean *r* = −0.26, 95% CI (−0.48 −0.04), *t*(32) = 2.4, *p* = 0.021, *d* = 0.42; ranking × rating: mean *r* = 0.84, 95% CI (0.76 0.91), *t*(32) = 22.8, *p* < 0.001, *d* = 4]. Average ratings for each category had a mean of 2.7 [95% CI (2.4 3.1)], and did not significantly differ from the majority of individuals [*t*(522) = 0.28, *p* = 0.78, *d* = 0.05]. Inverted thresholds have never been reported in laboratory cohorts (Abram et al., 2016, 2019a) or in rodents, but similar proportions of inverted individuals have been seen in other mTurk samples (Schmitzer-Torbert et al., 2018). While we cannot say why some individuals showed inverted choices, one potential explanation is that they were multi-tasking. We compared the proportion of inverted performances between versions, and found no significant difference [original version mean = 7.7% (20 participants), 95% CI (4.5 11.0); distractor version mean = 4.6% (13 participants), 95% CI (2.2 7.1); *χ*^2^(1, *N* = 539) = 2.22, *p* = 0.136, *φ* = 0.064].

Individuals showing inverted behavior were likely solving the task using a different strategy than those showing non-inverted behavior. As support for the idea that inverted behavior reflected an inverted strategy, we found that subjects showing inverted behavior also showed an inverted relationship between thresholds and average video ratings, but consistent relationships between category rankings and video ratings. Importantly, we can identify inverted behavior in a well-defined way as subjects in which the best-fit thresholds were inverted. We measured both typically-oriented and inverted thresholds in all subjects and took the best-fit. We have included the results of the inverted behavior in Supplemental Figure S1. Because this small subset of individuals behaved irrationally within the task structure, we removed individuals who showed inverted choices from further analyses.

**Figure 3:**
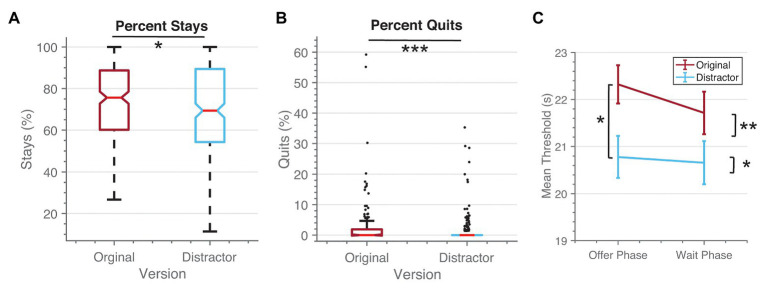
Distraction changed choices. **(A)** When the distractor was added, individuals chose to enter the Wait Phase significantly less than in the original version. **(B)** Once in the Wait Phase, individuals were significantly less likely to quit when the distractor was present. **(C)** When comparing thresholds calculated at the offer phase and the wait phase separately, we see that there is a significant difference between groups only in the Offer Phase. * <0.05, ** <0.01, *** <0.001

We found that including the distractor in the wait phase affected choices in both the offer and wait phases. In the offer phase, those playing the distractor version were less likely to choose to enter the wait phase compared to the original version [original version mean = 73.4, 95% CI (70.9 75.9); distractor version mean = 69.6, 95% CI (66.9 72.2); *t*(504) = 2.07, *p* = 0.038, *d* = 0.185]. However, once in the wait phase, individuals who completed the distractor task were significantly less likely to quit [original version mean = 3.1%, 95% CI (1.7 4.4); distractor version mean = 1.3, 95% CI (0.8 1.9); *t*(504) = 2.47, *p* = 0.014, *d* = 0.215; see Figure 3]. Additionally, we compared thresholds measured based on choices in the offer phase (before quits) and in the wait phase (after quits) to examine the impact of the distractor on the willingness to wait for the video clips. We found significant main effects and an interaction effect between phase and version [*version*: *F*(1, 504) = 4.3, *p* = 0.039, *η^2^* = 0.008; *phase*: *F*(1, 504) = 13.4, *p* < 0.001, *η^2^* = 0.001; *interaction*: *F*(1, 504) = 5.9, *p* = 0.015, *η^2^* = 0.0002]. Importantly, there was a significant difference between the versions only during the offer phase [*offer phase*: *t*(504) = 2.47, *p* = 0.014, *d* = 0.090; *wait phase*: *t*(504) = 1.63, *p* = 0.103, *d* = 0.016]. Finally, there was no significant difference in the rated enjoyment of the videos across the versions [original version: 2.7, 95% CI (2.6 2.8); distractor version: 2.7, 95% CI (2.5 2.8); *t*(489) = 0.49, *p* = 0.62, *d* = 0.045]. Performance on the distractor task additionally indicated improved consistency of decisions on the WebSurf task (see Supplemental Material and Supplemental Figure S2).

We measured sunk cost behavior in the task, comparing across the simple manipulation of whether participants were actively or passively waiting for the reward. Sunk cost was measured by calculating the probability that a video clip was earned for each offer value, based on the amount of time that had already been spent in the wait phase. An effect of sunk costs on decisions would be reflected by finding that as time spent in a trial increased, the probability of waiting out the delay to watch the video increased. We, therefore, examined the probability of earning the video as a function of the interaction of time remaining in the wait phase and time spent already waiting.

**Figure 4:**
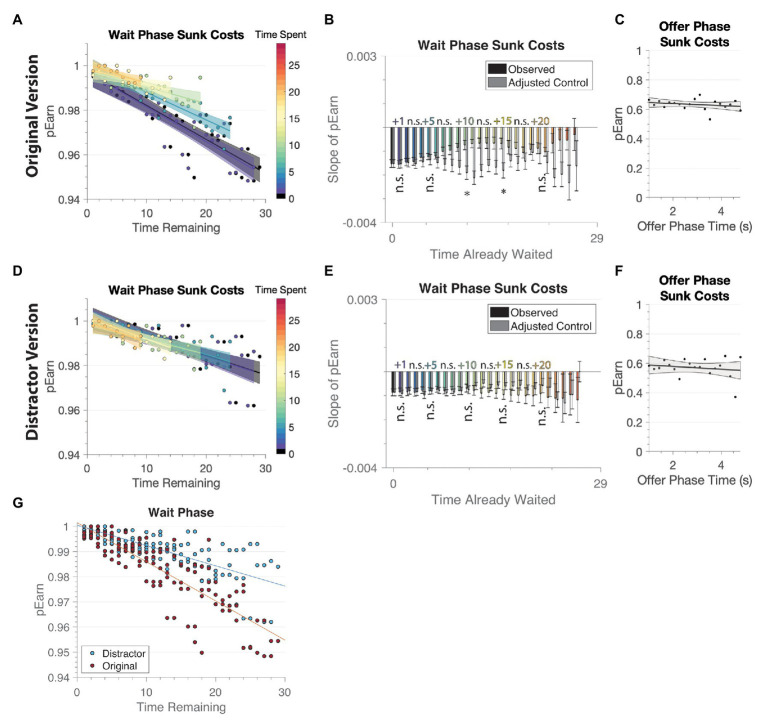
Sunk cost is not evident when the distractor is present. **(A,B,C)** Original Version. **(A)** We replicated sunk cost effects in the original version of the task, in which the longer an individual spent waiting, the more likely they would earn the final video instead of quitting. pEarn is the probability of waiting until the end to receive the reward. **(B)** A slope was calculated for each integer of time spent using a linear regression (slope of pEarn). To control for the differences in the number of observable data points for each of these lines, we compared the observed slope to an adjusted control, created by matching the values in time remaining at 0s spent to the observed time spent and taking the slope of those values. Error bars are ±1 SEM. **(C)** There was no relationship between earning the video and wait time in the offer phase. **(D,E,F)** Distractor Version. **(D)** When the distractor was present, we did not see any evidence of sunk cost. **(E)** Unlike the original version, there were no significant differences in the slopes of pEarn when the distractor was added. **(F)** Similar to the original version, there was no relationship between earning the video and wait time in the offer phase. **(G)** Here we simplify the comparison to show the interaction effect of Time remaining × Version. There is a greater slope in the original version, in which individuals were less likely to earn the reward the longer they had left to wait. This finding is consistent with overall more quits in the original version particularly early on during the wait phase (where time remaining was greater). * <0.05, ** <0.01, *** <0.001 after Bonferroni correction for multiple comparisons.

First, our results on the original version replicated the observation that subjects showed a susceptibility to sunk costs during the wait phase [two-way ANCOVA collapsing across all galleries. *Time spent*: *F*(1, 119) = 0.11, *p* = 0.74, *η^2^* = 0; *time remaining*: *F*(1, 119) = 299.3, *p* < 0.001, *η^2^* = 0.426; *interaction*: *F*(1, 119) = 26.7, *p* < 0.001, *η^2^* = 0.038]. Time spent and time remaining refer to the length of time in a single trial, measuring how much time a subject has spent waiting in a given trial and how much time a subject will have to spend before receiving the video reward, respectively. Thus, a main effect of time remaining was identified, in which the likelihood of earning a video increased as the amount of time left decreased. Importantly, there was a significant interaction effect, showing that while the amount of time decreased closer to the reward, having spent more time waiting increased the likelihood of waiting out the full delay to earn the video. Additionally, no significant effect of time spent in the wait phase was found, suggesting that this effect was not simply due to how much time was spent, but that the value of the time remaining interacted with the time spent. Moreover, again replicating previous studies, no significant effect of time spent in the offer phase was found (*r* = −0.25, *p* = 0.31; Figure 4C). This shows that our original observations are not specific to University of Minnesota undergraduate cohorts, but generalize to larger populations such as mTurk users.

This pattern of behavior was not found in the distractor version. Instead, the amount of time spent in the wait phase had no impact on the likelihood of waiting out the delay to watch the video [two-way ANCOVA collapsing across all galleries. *Time spent*: *F*(1, 119) = 0.77, *p* = 0.38, *η^2^* = 0.003; *time remaining*: *F*(1, 119) = 92.8, *p* < 0.001, *η^2^* = 0.325; *interaction*: *F*(1, 119) = 0.15, *p* = 0.70, *η^2^* = 0]. While time remaining was significantly associated with the probability of earning the video, there was no relationship with the amount of time spent nor a significant interaction (see Figures 4A,D). A three-way ANCOVA of time remaining, time spent, and condition (original or distractor version) revealed a significant three-way interaction [*F*(1, 238) = 12.62, *p* < 0.001, *η^2^* = 0.011], showing further support for a significant change in sunk cost behavior (time spent vs. time remaining) when the distractor was added. There was a significant interaction between time remaining and version [*F*(1, 238) = 39.12, *p* < 0.001, *η^2^* = 0.034], in which subjects playing the distractor version were less likely to quit regardless of time remaining, while individuals in the original version were more likely to quit when time remaining was high, but decreased quit behavior as time remaining decreased (Figure 4G). There was no significant interaction between the time spent and version [*F*(1, 238) = 0.12, *p* = 0.73, *η^2^* = 0]. There was no main effect of version [*F*(1, 238) = 2.35, *p* = 0.127, *η^2^* = 0.002].

To further test the hypothesis that increasing the time spent led to a greater probability of staying through the delay, we controlled for the amount of time remaining by limiting analyses to only trials that had the same amount of time remaining. For example, to compare the probability of earning the reward after 5s spent to when the participant first started (0s), we limited the 0s spent group to only observations to up to 25s left, thus providing an adjusted control. In this analysis, there was a significant difference between the observed and adjusted control after 10s spent only in the original version [original version: *time spent*: *F*(1, 44) = 0.32, *p* = 0.58, *η^2^* = 0.002; *time remaining*: *F*(1, 44) = 58.5, *p* < 0.001, *η^2^* = 0.454; *interaction*: *F*(1, 44) = 11.3, *p* = 0.002, *η^2^* = 0.265; distractor version: *time spent*: *F*(1, 44) = 0.73, *p* = 0.40, *η^2^* = 0.009; *time remaining*: *F*(1, 44) = 39.2, *p* < 0.001, *η^2^* = 0.526; *interaction*: *F*(1, 44) = 0.09, *p* = 0.764, *η^2^* = 0]. We used a similar method to compare between later time points. For instance, we truncated the number of time points in the 1s spent group in order to compare to the 5s spent group [original version: *time spent*: *F*(1, 44) = 1.13, *p* = 0.29, *η^2^* = 0.006; *time remaining*: *F*(1, 44) = 15.6, *p* < 0.001, *η^2^* = 0.085; *interaction*: *F*(1, 44) = 1.13, *p* = 0.29, *η^2^* = 0.006; distractor version: *time spent*: *F*(1, 44) = 0.11, *p* = 0.74, *η^2^* = 0; *time remaining*: *F*(1, 44) = 9.36, *p* = 0.004, *η^2^* = 0.08; *interaction*: *F*(1, 44) = 0.63, *p* = 0.43, *η^2^* =0.006]. The results of each comparison are noted in Figures 4B,E; as is evident in the figure, none of these comparisons were significant in the distractor version, but the difference in the original version reflected a large change at the 5s time point. The only significant difference between the times spent was comparing 1 to 5s; furthermore, there were no significant differences between the adjusted control and the observed data until 5s. Finally, the distractor version also did not show sunk cost during the offer phase, which confirms that sunk costs only start to accrue after commitment to a decision, not simply the passage of time overall (distractor: *r* = −0.16, *p* = 0.5; Figure 4F).

**Figure 5:**
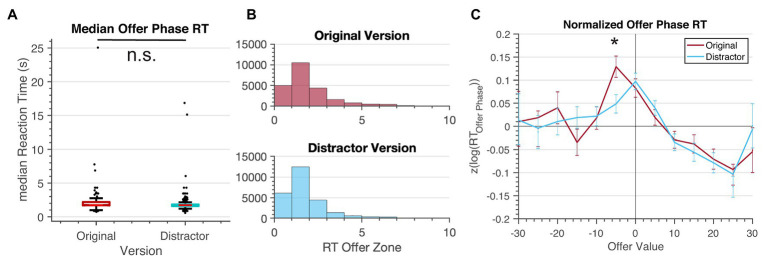
Offer Phase Reaction Times were short, despite unlimited time. **(A)** There was no significant difference in the median reaction time during the offer phase across the versions. **(B)** Histogram of reaction time during the offer phase for the original version and distractor version, truncated to show the majority of decisions (~92%). **(C)** Log offer phase reaction time (normalized for each individual) compared to the offer value (threshold – offer time amount) showed increased reaction time in slightly-worse-than-threshold offers, but only for the original version. * <0.05, ** <0.01, *** <0.001.

Those who showed inverted behavior should theoretically not show the sunk cost behavior, as they prefer waiting. When we analyze the inverted samples only, they do not show the sunk cost effect in either game (see Supplemental Figure S3). To assess the impact of the distractor task on cognitive processes, we additionally looked at the reaction time during the offer phase. For the original version, median reaction time was 1.73s, with a maximum of 286s (skewness = 24.0, kurtosis = 771). For the distractor version, median reaction time was 1.73s with a maximum of 289s (skewness = 18.2, kurtosis = 503). We compared these two samples using a Wilcoxon’s Rank Sum which identified significant differences between the two samples (*W* = 6.04 ×10^8^, *p* < 0.001). When comparing median reaction time for each participant across groups, we found no significant difference [*t*(504) = 0.97, *p* = 0.33, *d* = 0.086; Figure 5A,B]. Additionally, when comparing normalized offer phase reaction time as a function of the offer value (threshold – offer time amount), we found that decisions were most difficult (i.e., longest reaction time) closest to the threshold. Additionally there was an increased reaction time for options that were slightly worse than the threshold, as seen in earlier studies (Sweis et al., 2018d), in the original version compared to the distractor version [*Z*-score offer phase reaction time – original: 0.129, 95% CI (0.084 0.174); distractor: 0.049, 95% CI (0.010 0.088); *t*(504) = 2.48, *p* = 0.013, *d* = 0.079]. However, this finding did not survive Bonferroni correction (*α*/13 = 0.004). All other bin comparisons across version were not significantly different.

## Discussion

The sunk cost fallacy arises when subjects make decisions based on already spent costs rather than on the expected future earnings (Staw and Ross, 1989). In a situation where the expected future earning is completely cued (as in the WebSurf task), making decisions based on effort already spent is economically irrational. In previous studies using the WebSurf task and its rodent-equivalent Restaurant Row, sunk costs were evident in mice, rats, and humans in that time spent waiting in the wait-phase impacted decisions to quit (Sweis et al., 2018a). However, across all three species, sunk costs were not evident during the offer phase, suggesting that sunk costs only started to accrue after commitment to a decision. Our original version, matching the original study, replicated these findings. However, when we added the attention-check in an attempt to prevent multi-tasking, we found that subjects no longer showed any evidence of including sunk costs in their decisions, indicated by the lack of interaction between time spent and time remaining in the wait phase.

Instead, we found that individuals committed more to the offer during the offer-phase, rather than re-evaluating decisions in the wait-phase. Participants that experienced the distractor task were less likely to enter the wait phase. However, once they were in the wait phase, they were less likely to quit, showing more commitment to the reward. One possibility is that the attention-check served as a distractor, making the subject less aware of the passage of the delay, but also making the overall waiting process more aversive, evidenced by the fact participants were more likely to skip out of the offer phase in the distractor version. In particular, we observed that a switch in behavior occurred after ~5s spent in the original version, in which participants became far less likely to quit. This switch may indicate a change from a deliberative state, in which the participant is still assessing the decision to wait for a short period of time after entering the wait phase, to an implemental state, in which the participant has committed to the engaged task (Gollwitzer, 2012). In contrast, we found no evidence of a change in behavior once entering the wait phase for the distractor version.

Within the offer phase itself, reaction times are longer for skips than stays, implying a default option that is likely over-ridden, inconsistent with the rational theory that subjects should spend their decision-time cost within the wait phase, and consistent with other experiments in humans on WebSurf (Abram et al., 2016) and rodents (rats, mice) on Restaurant Row (Wikenheiser and Redish, 2012; Steiner and Redish, 2014; Sweis et al., 2018a). Within the wait phase, we did find evidence for re-evaluation that depended on sunk costs, but that dependence disappeared in the distractor version. This suggests that the re-evaluation and its sensitivity to sunk costs likely depends on experiencing (and attending to) the cost. In some instances, an individual may initially have a higher threshold, but after experiencing the cost (i.e., wait time) they may change their mind, thus “quitting” out of a stay decision.

Subjects playing the distractor version showed a decreased willingness to quit and decreased sunk costs. One possibility is that the distractor required additional cognitive effort towards a different goal, which decreased executive processing availability to re-evaluate the decision once in the waiting phase. The focus on an alternate task may have decreased the salience of the passage of time, the sunk cost in this experiment, vs. more acute awareness of the passage of time during the more boring wait phase in the original version (Danckert and Allman, 2005). The addition of the distractor task may therefore push individuals into a more implemental (goal-oriented, committed) mindset compared to a deliberative (open-minded, future-considering) mindset, which would lead to fewer re-appraisals (and therefore fewer quits; Gollwitzer, 2012). Similarly, the offer phase would also likely involve additional cognitive effort to make a decision, which may explain why sunk costs are not evident during those decisions.

Quitting out of the wait phase is actually rational in the Websurf task. It is the time spent in the offer phase that is irrational. If we hypothesize a non-zero decision time (i.e., it takes some non-zero time to process the gallery, the delay, and the internal willingness-to-wait), then the optimal process is to choose “STAY” as quickly as possible and to evaluate the decision while the wait phase delay is counting down, quitting if the remaining wait time is greater than the subject’s threshold for that gallery. If subjects were doing this, they would show very fast offer phase responses and the time spent before quitting would be a constant. In practice, this behavior is actually quite rare, which begs the question of why people are willing to spend time deciding, yet not waiting. As we have shown, these two decision points get treated differently, and sunk cost is only seen in the wait phase, but not the offer phase, in the original version. In fact, as pointed out in previous studies (Sweis et al., 2018a), one explanation for the fact that most subjects make their decisions in the offer phase is that they are avoiding the sunk costs that normally accrue in the wait phase. Interestingly, the distractor-version subjects avoided the wait phase even more than the original-version subjects.

The use of an intervention to change states is not unique. Reimer et al. (2010) suggested that group members were more able to solve a hidden profile problem, which is impacted by prior preference, when shifted towards a deliberative mindset. For example, asking subjects to plan to deliberate using if-then reasoning leads to increased use of deliberative processes, more than merely stating the intention to deliberate. This action-oriented implementation has been more effective than simply stating intentions to be more balanced in decision making (Thürmer et al., 2015; Wieber et al., 2015; Doerflinger et al., 2017). In the distractor task in our study, each button press in the distractor task might have been seen as a “sub-goal,” keeping individuals in a goal-directed state and therefore changing their responses to the sunk costs expended in the delay. However, we find this unlikely because the response required in the distractor had to be made only when the attention-check button changed color, making it unlikely to be a planned subgoal, although it remains possible that subjects may have been in an action-prepared state during the distractor task, which could have distracted them from the countdown during the wait phase, making behavior unrelated to the sunk costs already expended.

An alternate hypothesis is that participants lose “inertia” during the original version of the task, as they are simply waiting without any other focus except for the passage of time. However, if participants simply disengaged from the task (i.e., lost inertia) we would anticipate that they would quit less in the original version. Instead, we found the opposite effect, in which those engaged in the distractor task chose to quit less. Those that were simply waiting (in the original version) were on average were more likely to leave the wait phase when it was not valuable to them, suggesting a re-evaluation process (Sweis et al., 2018a).

It is possible that waiting out the delay was particularly boring and the attention-check in the distractor task provided a mini-game that reduced boredom. However, in order to explain our data, we would have to hypothesize that this change also reduced the general process that was driving the sensitivity to sunk costs. This hypothesis that a sensitivity to time-related sunk costs is related to developing boredom is akin to ideas about explanations for cognitive task switching (Shenhav et al., 2017) and is an interesting hypothesis to be pursued in future studies. However, the distractor-version subjects avoided the wait phase even more than the original-version subjects, implying that the distractor-version was aversive not attracting.

In mice on the analogous Restaurant Row task, optogenetic alteration of the strength of synaptic transmission between the infralimbic cortex to nucleus accumbens shell led to a decrease in the willingness to quit from the wait phase and to a greater effect of sunk costs; initial commitment decisions in the offer phase were not affected (Sweis et al., 2018b). Sweis et al. (2018b) hypothesized that re-evaluation depended on a cognitive override from the frontal cortex. When compared to our findings, this may indicate that if the frontal cortex is preoccupied with another cognitive task, it will not be able to engage in that re-evaluation of the decision to wait or quit. The additional cognitive load might not have allowed subjects to engage the necessary attention to the task needed to quit subpar encounters.

We originally included the distractor in order to increase task engagement and particularly to reduce the number of inverted patterns of behavior. However, this small adjustment had significant impacts on task behavior elsewhere, including reducing the number of quits and overall sensitivity to sunk cost. This shift in behavior online speaks to the efficacy of testing participants outside the laboratory environment. Clearly, some portion of individuals behave differently than if they were participating in a laboratory experiment. However, only a small number of individuals (6% across the samples) showed this inverted shift in behavior. The addition of the distractor task had limited effect in reducing the inverted behavior, but had a strong effect on the sunk cost behavior. These findings illustrate the need to be cautious in assessing task engagement when we utilize online participants (Hauser et al., 2018).

It is also possible that individuals in this study were not motivated by the short video clips, but instead by payment for completion of the task with minimal engagement. As seen in Figure 2, the amount of time willing to wait for a reward is strongly related to the rated satisfaction of a video. The overwhelming majority of individuals engaged with the task in a similar way as rodents do for food that they enjoy and to undergraduates who are not being paid money (Steiner and Redish, 2014; Abram et al., 2016; Sweis et al., 2018d). We suggest instead that individuals were seeking out videos they would enjoy. This result suggests that in an economy of time, individuals are generally still trying to maximize the amount of enjoyment (i.e., watching videos they like) during the time that they are engaged in the study. The benefit of WebSurf task is that it is flexible to allow individuals to seek out their own preferences; while one person may prefer kitten videos, another may prefer bike accident videos. The average ratings of videos were similar to past samples, and we tried to mitigate inactivity by removing participants who were inactive for an unusually long time. Moreover, the majority of trials had a reaction time <5s, indicating that individuals treated the task like other experiments that have a time limit (Figure 5). Furthermore, stipulations in the game require that participants engage with the task; they cannot simply wait and do nothing the entire 30min. When we had undergraduates in the lab complete this task, with an experimenter present, they also chose to spend their time watching videos. Killing time might be a possible rationale for the inverted results; perhaps it is preferable for a subset of individuals to do nothing over watching a short video, which we can still identify based on the choices made about an investment of time.

A portion of individuals started the task, but did not complete it. Due to the remote nature of the experiment, we do not have full information about the reasons for this behavior, particularly whether it might have been due to boredom, distraction, or technical difficulties. In addition, significantly more individuals prematurely withdrew from the study in the original version than the distractor version. Due to the lack of information on why these people quit the study, there may be some aspect of the study or the group of individuals that led to differences in outcomes. But more importantly, we found that our attempts to monitor attention had unexpected consequences on behavior. To better assess attention during the WebSurf task, tools like eye-tracking or mouse tracking could be valuable both for assessing task engagement and allowing experiments to understand what may be drawing the attention of the participants. Future experiments could include video recordings to observe not only visual engagement but also facial expressions that may indicate enjoyment. Adjusting the task to use mouse tracking may also help understand what individuals are focused on during the task, to know what mechanism may be driving changes in behavior across the two versions.

Finally, there are a few open-ended alternative explanations in our dataset. It is possible that we do not have enough variance in our data to observe escalation in quitting behavior over time in the distraction version, as fewer people chose to stay in the first place and then also quit less frequently. While this difference is true, there is enough data to detect a sensitivity to sunk costs in the distractor version. We also see that the data are not noisier, but rather aligned across Time Spent × Time Remaining (see Figure 4). Additionally, we collected our samples at two separate time points, ~3months apart. While participants were not randomly assigned to the different tasks, they were told the same advertisement that they would be playing a game in which they would make choices to watch and rate short video clips. We have little reason to believe that these samples would be particularly distinct. Indeed, our demographic information is generally consistent across each group.

Classic theories of sunk cost are based on hypotheses of cognitive dissonance (not wanting to see oneself as having made a mistake; Staw and Fox, 1977; Arkes and Blumer, 1985; Arkes, 1996), loss aversion (Kahneman et al., 1991; Gupta, 2009), avoidance of regret (recognizing mistakes of one’s own agency; Bell, 1982; Zeelenberg et al., 1998; Steiner and Redish, 2014; Sweis et al., 2018d), or social cues (concern about others’ recognition of one’s mistake; Kanodia et al., 1989; Staw and Ross, 1989; Arkes and Ayton, 1999), or interactions of these various components (Mcafee et al., 2010; Sleesman et al., 2012). Non-human animals have also been found to show a susceptibility to sunk costs (Dawkins and Brockmann, 1980; Aw et al., 2011; Pattison et al., 2012; Magalhães and White, 2016), and typical explanations of non-human sunk cost behaviors are based on increased energy needs that occur with effort spent, thus increasing the value of food rewards (Kacelnik and Marsh, 2002; Pompilio et al., 2006; Aw et al., 2011) or changes in the increasing contrast between the approach reward state and the current needful state (Singer and Zentall, 2011). Finally, some theories of sunk cost have suggested a dependence on mindset approaches to decisions before or after commitment (deliberative vs. implemental; Gollwitzer, 2012), which parallels findings that mechanisms differ depending on the type of decision (opt-out or binary choice) related to commitment to an action (Sweis et al., 2018c).

Our task varies somewhat from many other sunk cost paradigms. Some studies provide vignettes of an investment project, in which subjects have already invested a large or small amount of money (Staw, 1976, 1981; Arkes and Blumer, 1985; Whyte, 1993). For example, the Arkes and Blumer (1985) study used a short description of a ski trip, in which the participant learns that they purchased two tickets for the same day that vary in price and location. Participants were told that they would enjoy the cheaper one more, yet the majority of individuals still chose the more expensive trip. Many of these studies rely on a description of investments and outcomes. However, there are some advantages to studying sunk costs in the WebSurf paradigm.

An economy of time is a finite resource that cannot be spent or traded outside of the experiment. Spending costs from a limited temporal budget also provides for cross-species translation, as time is a primary resource and does not require assumptions about secondary reinforcers (like money). Similarly, the videos are a reward that is consumed within the task itself. While it is possible that such consumption can produce satiation, similar effects would be expected from food rewards (in rodents), and we have not seen strong evidence for satiation effects in either the WebSurf or Restaurant Row paradigms. Furthermore, the WebSurf task measures the individual preferences of the subject (in the choices of the galleries), allowing the subjects to reveal their preferences, which means that we do not need to assume imposed preferences on subjects. Individuals can determine and work towards their own preferences, instead of preferences laid out by the experimenter. In contrast, in the Arkes and Blumer (1985) ski-trip example, individuals may have still responded with their own preference, even if they were told what their preference “should” be. Finally, classic story-based (monetary) sunk costs paradigms do not separate deliberative and implemental decisions, as these stories provide for only a single choice to make. Separation of the offer and wait phases in the WebSurf task allow us to compare decision making and commitment to an outcome, and importantly, we find different sensitivities to sunk costs in the two phases.

However, it is important to note that the “effort” spent in our task is distinct from that in the other paradigms; namely, in the WebSurf task, the economic cost spent is that of time, while most other tasks use money as their economically limited resource. Other studies have also examined the impact of physical or mental effort made towards a desired reward (Clement et al., 2000). These different types of effort may have different mechanisms and caution should be taken when generalizing results across different economically limited resources. Our results support a change in mindset going from the offer phase to the wait phase, and that distraction during the wait phase reduces re-evaluation processes and more quickly solidifies the shift to an implemental mindset. While it is possible that the increased effort during the wait phase may make staying appear more valuable, we did not see an increase in the overall ratings of the videos, which would be a sign of cognitive dissonance (Festinger aand Carlsmith, 1959), unlike the risk variant of WebSurf, in which a surprising increase in delay did increase overall ratings (Abram et al., 2019b). Hypotheses related to loss aversion and avoidance of regret do not specifically incorporate the impact of a distractor. However, if anything, a distractor should increase the cost of delay, which in turn should increase loss aversion and potential regret; these hypotheses are incongruent with our observation that the distractor decreased sunk costs. While social cues were not explicitly controlled in this experiment, it is unlikely that participants were making mTurk decisions based on social cues. As we do not use food rewards, it is unlikely that watching short video clips would replenish energy needs, and thus our results do not support the non-human animal hypotheses regarding increased energy needs. Our finding that sunk costs are not evident in the offer phase, which replicates previous studies, additionally counters hypotheses that sunk costs are linearly related to effort, regardless of commitment towards the reward.

Our data indicate that attention is a key element of sunk costs; when one fails to attend to the loss of time, one is less likely to quit a subpar choice. Simple distraction from re-evaluation of a decision may indicate a change in the mindset of the decision, i.e., one that is open for reconsideration or one that is focused on achieving the goal of the outcome. Future studies of sunk cost should consider the impact of attention to the task during decisions, as this cognitive ability seems to play an important function in the sunk cost effect and in the re-evaluation of decisions.

## Data Availability Statement

The datasets presented in this study can be found in online repositories. The names of the repository/repositories and accession number(s) can be found at: https://osf.io/v3e8t/.

## Ethics Statement

The studies involving human participants were reviewed and approved by Institutional Review Board, University of Minnesota. The patients/participants provided their written informed consent to participate in this study.

## Author Contributions

Task design: RK, ADR, AWM. Task implementation: RK. Data analysis: RK with ADR and AWM supervision. All authors contributed to the article and approved the submitted version.

### Conflict of Interest

The authors declare that the research was conducted in the absence of any commercial or financial relationships that could be construed as a potential conflict of interest.
