# Supplementary material for: Sensitivity to Sunk Costs Depends on Attention to the Delay
Source: Front Psychol. 2021 Feb 22;12:604843. doi: 10.3389/fpsyg.2021.604843 (PMC7937795; doi:10.3389/fpsyg.2021.604843)
Supplement: Supplementary file 1 [file Presentation_1.pdf]

Sensitivity to sunk costs depends on attention to the delay

Rebecca Kazinka[1], Angus W. MacDonald[2], & A. David Redish[3]

1. Graduate Program in Clinical Science and Psychopathology Research, Psychology Department, University of Minnesota
2. Psychology Department, University of Minnesota
3. Neuroscience Department, University of Minnesota

Supplemental Methods

D' was calculated by the difference between the proportion of true positives and false positives. As the color change could happen several times in a trial (depending on the length), true positives were calculated as the number of correct responses when the button changed color. False positives were identified as a click outside of the color change as a binary outcome, even if a participant clicked several times within the time window. If the timer ended during a color change, this response was not counted, as they did not have adequate time to respond.

Supplemental Analyses

We examined the sunk cost effect in the inverted sample for both versions. We had significantly fewer individuals in these groups, and therefore it is more difficult to make an assessment of the sunk cost effect statistically. However, based on the results, we do not see the sunk cost effect in either version (Figure S3). In the original version, there was a main effect of time remaining, yet no main effect of time spent or interaction of time spent and time remaining in the trials (two-way ANOVA collapsing across all galleries. *time spent*: $F(1, 119) = 0.59$, $p = .444$, $\eta^2 = .004$; *time remaining*: $F(1, 119) = 10.61$, $p = .002$, $\eta^2 = .079$; *interaction*: $F(1, 119) = 0.31$, $p = .582$, $\eta^2 = .002$). Similarly, there was a main effect of time remaining, but not time spent nor interaction of time spent and time remaining in the distractor version (two-way ANOVA collapsing across all galleries. *time spent*: $F(1, 119) = 1.76$, $p = .187$, $\eta^2 = .012$; *time remaining*: $F(1, 119) = 30.16$, $p <. 001$, $\eta^2 = .187$; *interaction*: $F(1, 119) = 1.97$, $p = .163$, $\eta^2 = .012$). When comparing the versions, there was a marginally significant main effect of version ($F(1, 238) = 3.42$, $p = .066$, $\eta^2 = .014$); additionally, there was a marginally significant interaction of time remaining and version($F(1, 238) = 3.35$, $p = .065$, $\eta^2 = .014$). As expected, there was a main effect of time remaining ($F(1, 238) = 39.01$, $p < .001$, $\eta^2 = .164$). All other effects were not significant.

Supplemental tables and figures

	Original Version			Distractor Version		
	N	Gender M/F/N (%)	Age Mean (SD)	N	Gender M/F/N (%)	Age Mean (SD)
Started	651	-	-	579	-	-
Completed Questionnaire	583	48/52/<1	35.3(10.6)	531	50/49/<1	36.8 (11.1)
Passed Bot Check	423	45/54/<1	36.5 (11.2)	381	50/49/<1	37.8 (11.2)
Completed all tasks	259	47/53/<1	36.6 (11.6)	280	50/49/1	38.1 (11.3)

Table S1. Summary of demographics. We show the breakdown the number of individuals collected at each stage of the HIT: started the HIT, successfully passed the bot check, and met eligibility criteria for the WebSurf task. We further separate out eligibility, gender, and age by the two different tasks, the original version and the distractor version. Because of the placement of the demographic questions, we do not have gender and age information for those who began the questionnaire but did not complete it.

Supplemental Figure S1. Inverted participants showed a reversal in performance. A-C. A small subset of individuals showed “inverted” responses, i.e. stayed for longer delays and skipped shorter ones (A). In contrast to the expected pattern, these thresholds are negatively correlated with ratings (B) and rankings (C). * <.05, ** <.01, *** <.001

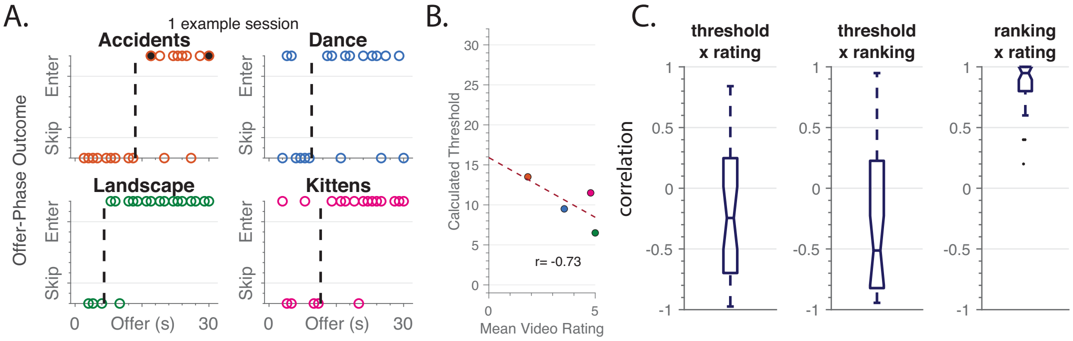
A.
1 example session
Accidents
Dance
Landscape
Kittens
Offer-Phase Outcome
Enter
Skip
0
Offer (s)
30
B.
Calculated Threshold
Mean Video Rating
r= -0.73
C.
threshold x rating
threshold x ranking
ranking x rating
correlation

Supplemental Figure S2. Attention affected performance. Those that were less accurate on the distractor task (calculated with d-prime) were more likely to have high inconsistency on trial choices. This finding suggests that the distractor task, which was a simple attention task, identified those that were paying attention to the task overall.

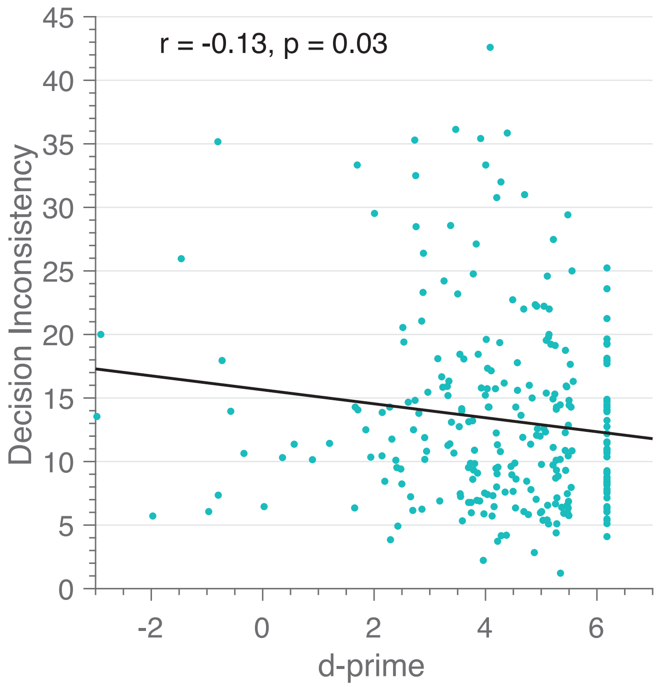
r = -0.13, p = 0.03
Decision Inconsistency
d-prime
0
5
10
15
20
25
30
35
40
45
-2
0
2
4
6

Supplemental Figure S3. Individuals with inverted behavior did not show the sunk cost effect in either version. A-C. Original Version. A. There was a significant interaction of time remaining, in which there were fewer quits when the time remaining was shorter, but no evidence of sunk cost sensitivity. pEarn is the probability of waiting until the end to receive the reward. B. A slope was calculated for each integer of time spent using a linear regression (slope of pEarn). We compared the observed slope to an adjusted control slope by matching the values in time remaining at 0 s spent, thus controlling for the differences in the number of observable data points. We found no significant differences between the adjusted control or the observable data. Error bars are±1 SEM. C. There was no significant relationship between earning the video and time spent in the offer phase. D-F. Distractor Version. Similarly, there was a significant interaction of time remaining yet no evidence of the sunk cost effect. E. There were no significant differences between the adjusted control and the observable data. Additionally, there were not enough quits when more time was spent to calculate the slopes (denoted with n.a.). F. There was not a significant relationship between time waited in the offer phase and probability of earning the video.

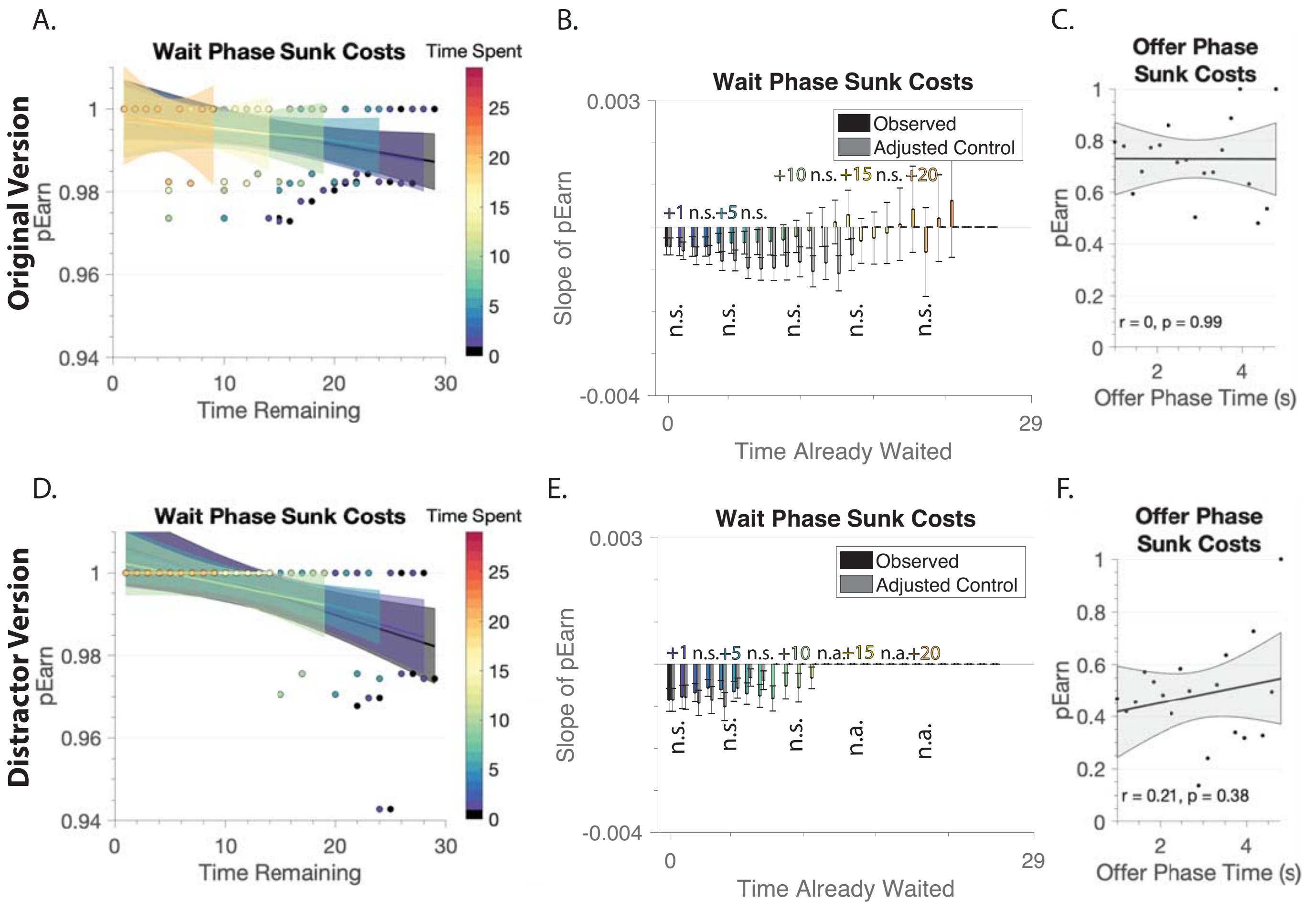
A.
Original Version
Wait Phase Sunk Costs
Time Spent
pEarn
Time Remaining
B.
Wait Phase Sunk Costs
Observed
Adjusted Control
Slope of pEarn
Time Already Waited
+1 n.s. +5 n.s.
+10 n.s. +15 n.s. +20
n.s.
C.
Offer Phase Sunk Costs
r = 0, p = 0.99
Offer Phase Time (s)
D.
Distractor Version
Wait Phase Sunk Costs
E.
+1 n.s. +5 n.s. +10 n.a. +15 n.a. +20
n.a.
F.
r = 0.21, p = 0.38

	Original Version			Distractor Version		
	N	Gender M/F/N (%)	Age Mean (SD)	N	Gender M/F/N (%)	Age Mean (SD)
Started	651	-	-	579	-	-
Completed Questionnaire	583	48/52/<1	35.3(10.6)	531	50/49/<1	36.8 (11.1)
Passed Bot Check	423	45/54/<1	36.5 (11.2)	381	50/49/<1	37.8 (11.2)
Completed all tasks	259	47/53/<1	36.6 (11.6)	280	50/49/1	38.1 (11.3)

Table S1. Summary of demographics. *We show the breakdown the number of individuals collected at each stage of the HIT: started the HIT, successfully passed the bot check, and met eligibility criteria for the WebSurf task. We further separate out eligibility, gender, and age by the two different tasks, the original version and the distractor version. Because of the placement of the demographic questions, we do not have gender and age information for those who began the questionnaire but did not complete it.*
